# Twins with temporal lobe epilepsy: genetic contributions to hippocampal sclerosis and other subtypes

**DOI:** 10.1093/brain/awaf209

**Published:** 2025-06-18

**Authors:** Yew Li Dang, Kate Esnault, Gregory Fitt, Terence J O’Brien, Graeme D Jackson, Piero Perucca, Samuel F Berkovic

**Affiliations:** Epilepsy Research Centre, Department of Medicine (Austin Health), The University of Melbourne, Melbourne, Victoria 3084, Australia; Bladin-Berkovic Comprehensive Epilepsy Program, Department of Neurology, Austin Health, Melbourne, Victoria 3084, Australia; Epilepsy Research Centre, Department of Medicine (Austin Health), The University of Melbourne, Melbourne, Victoria 3084, Australia; Bladin-Berkovic Comprehensive Epilepsy Program, Department of Neurology, Austin Health, Melbourne, Victoria 3084, Australia; Department of Radiology, Austin Health, Melbourne, Victoria 3084, Australia; Department of Neuroscience, School of Translational Medicine, Monash University, Melbourne, Victoria 3004, Australia; Department of Neurology, Alfred Health, Melbourne, Victoria 3004, Australia; Department of Neurology, The Royal Melbourne Hospital, Melbourne, Victoria 3050, Australia; Bladin-Berkovic Comprehensive Epilepsy Program, Department of Neurology, Austin Health, Melbourne, Victoria 3084, Australia; Neuroimaging and Neural Networks, The Florey Institute of Neuroscience and Mental Health, Melbourne, Victoria 3052, Australia; Epilepsy Research Centre, Department of Medicine (Austin Health), The University of Melbourne, Melbourne, Victoria 3084, Australia; Bladin-Berkovic Comprehensive Epilepsy Program, Department of Neurology, Austin Health, Melbourne, Victoria 3084, Australia; Department of Neuroscience, School of Translational Medicine, Monash University, Melbourne, Victoria 3004, Australia; Department of Neurology, Alfred Health, Melbourne, Victoria 3004, Australia; Department of Neurology, The Royal Melbourne Hospital, Melbourne, Victoria 3050, Australia; Epilepsy Research Centre, Department of Medicine (Austin Health), The University of Melbourne, Melbourne, Victoria 3084, Australia; Bladin-Berkovic Comprehensive Epilepsy Program, Department of Neurology, Austin Health, Melbourne, Victoria 3084, Australia

**Keywords:** twin study, temporal lobe epilepsy, hippocampal sclerosis, genetics, neurofibromatosis

## Abstract

Temporal lobe epilepsy is the most common focal epilepsy in adults. While temporal lobe epilepsy was historically perceived to have a largely acquired aetiology, growing evidence points to important genetic contributions. There are several temporal lobe epilepsy subtypes, including mesial temporal lobe epilepsy with or without hippocampal sclerosis, but the relative genetic contributions to each of these subtypes have not been directly studied.

In this study, we use the classical twin model in 80 twin pairs where at least one twin had temporal lobe epilepsy. We assessed the genetic contribution to various subtypes [lesional temporal lobe epilepsy, non-lesional temporal lobe epilepsy, mesial temporal lobe epilepsy (with or without hippocampal sclerosis), lateral temporal lobe epilepsy and non-localized temporal lobe epilepsy], by analysing the concordance for temporal lobe epilepsy in monozygotic twins compared with dizygotic twins. In the 10 monozygotic pairs where at least one twin had hippocampal sclerosis, we searched for within-pair acquired differences between affected and unaffected individuals.

There was an excess of monozygotic pairs concordant for temporal lobe epilepsy compared with dizygotic pairs (17/47 concordant monozygotic versus 0/33 concordant dizygotic, *P* < 0.05). This supports a genetic contribution to temporal lobe epilepsy, but notably this concordance was driven by non-lesional temporal lobe epilepsy cases, particularly mesial temporal lobe epilepsy without hippocampal sclerosis (14/22 concordant monozygotic versus 0/11 concordant dizygotic, *P* < 0.05). No concordant monozygotic or dizygotic pairs were observed in the lesional temporal lobe epilepsy (*n* = 8) and non-localized temporal lobe epilepsy (*n* = 15) groups.

The concordance for temporal lobe epilepsy in monozygotic twins with mesial temporal lobe epilepsy with hippocampal sclerosis was much lower (2/10 concordant monozygotic versus 0/9 concordant dizygotic, *P* = 1), suggesting a lesser contribution from germline genetic causes to mesial temporal lobe epilepsy with hippocampal sclerosis. Eight monozygotic twin pairs were discordant for hippocampal sclerosis. In four of these pairs, both twins had febrile seizures, but hippocampal sclerosis was only present in the twin who had prolonged seizures.

The two monozygotic twin pairs concordant for hippocampal sclerosis had clinical neurofibromatosis type 1 with pathogenic germline *NF1* variants.

Our findings confirm a germline genetic component in temporal lobe epilepsy, strongest in mesial temporal lobe epilepsy without hippocampal sclerosis and present in lateral temporal lobe epilepsy but absent in lesional and non-localized temporal lobe epilepsy. In our mesial temporal lobe epilepsy with hippocampal sclerosis twins, we found both genetic factors (*NF1*) and prolonged febrile seizures contributed to the aetiology of hippocampal sclerosis.

## Teaser

Dang *et al*. use a classical twin study design to assess genetic contributions to different temporal lobe epilepsy (TLE) subtypes. Their findings suggest a strong germline genetic component in mesial TLE without hippocampal sclerosis, whereas both genetic factors and prolonged febrile seizures contribute to hippocampal sclerosis.

## Introduction

Temporal lobe epilepsy (TLE) is the most common focal epilepsy in adulthood.^1^ TLE can be stratified into various subtypes based on aetiology and predominant seizure semiology. Broadly, TLE can be divided into non-lesional TLE and lesional TLE (where an epileptogenic lesion is identified in the temporal lobe). These lesions include tumours, vascular malformations, malformations of cortical development and areas of gliosis. TLE can be further subclassified into mesial and lateral TLE subtypes, which are distinguished by their clinical presentations. Lateral TLE is associated with auditory auras and receptive aphasia, whereas mesial TLE (MTLE) is characterized by features suggesting a mesial onset of seizures, such as déjà vu, rising epigastric sensation and olfactory auras. MTLE can be familial (FMTLE), typically presenting with mild TLE without hippocampal sclerosis (HS) and characterized by well-controlled seizures.^2^ In contrast, MTLE associated with HS (MTLE-HS) is typically drug resistant. In this paper, we address the relative genetic contributions of each of these TLE subtypes, which have not previously been directly studied.

Although TLE has historically been regarded as a largely acquired disorder, familial forms have been described, and TLE is now recognized to have significant genetic contributions.^3-5^ The analysis of twin pairs is a powerful method to unravel the genetic architecture of complex disorders such as epilepsy, although it is not sensitive to somatic mosaicism. Twin studies provide evidence for a genetic basis for TLE by demonstrating higher concordance for TLE among monozygotic (MZ) compared with dizygotic (DZ) twins.^6,7^ Compared with lesional TLE, non-lesional TLE has been shown to have a higher heritable genetic component, suggesting distinct differences in germline genetic contribution between these two subtypes.^6,8^

In this study, we aimed to: (i) explore the genetic contributions to TLE and its various subtypes, including MTLE-HS, by comparing concordances for TLE and HS, respectively, in MZ and DZ twins; and (ii) identify non-genetic factors that may contribute to HS in MZ twins discordant for HS by applying a discordant MZ twin analysis, where MZ twins who are discordant for HS are compared.

## Materials and methods

### Ascertainment

Twin pairs with seizures were ascertained from community-based twin registers as well as referrals since 1988.^9^ Twins were mostly referred by physicians throughout Australia, with a smaller proportion of self-referred twins.

We examined 80 twin pairs with TLE; 54 of whom have been reported previously.^2,6,7,10-12^ Whereas previous reports had focused mainly on specific TLE subtypes, such as FMTLE cases, our current cohort included additional, newly ascertained twin pairs and explored the genetic contribution to TLE more broadly, including all of its currently recognized subtypes [lesional TLE, non-lesional TLE, mesial TLE (with or without HS), lateral TLE and non-localized TLE]. For the previously published pairs, all available relevant medical data were reviewed, and where possible, the twins were re-contacted to obtain further history and investigations. This allowed us to reclassify them based on the 2022 International League Against Epilepsy (ILAE) Classification of Epilepsy Syndromes.^13^

This study was approved by the Human Research Ethics Committee of Austin Health, Melbourne, Australia (H2007/02961). Written informed consent was obtained from all participating twins and their guardians in the case of minors.

### Clinical assessment

A comprehensive clinical evaluation of the twin pairs was undertaken. This included a structured interview with a validated questionnaire,^14^ physical examinations where possible, and a review of EEG and neuroimaging data. Relevant medical records including hospital admissions, neurology letters and results were reviewed. The questionnaire included seizure history, investigations, treatments, antenatal, birth and early developmental history, educational history, general medical history and family history. Informant interviews were conducted where required to corroborate details and aid in diagnosis.

### Seizure and epilepsy classification

The twin pairs were reviewed by three neurologists (Y.L.D., P.P., S.F.B.), to achieve consensus on the classification of epilepsy types and syndromes according to the 2017 ILAE Classification of the Epilepsies^15^ and the 2022 ILAE Classification of Epilepsy Syndromes.^13^

Only twins where at least one individual had confirmed TLE were included. The diagnosis of TLE was made based on clinical findings and concordant investigations such as EEG and neuroimaging supporting the diagnosis.

Affected probands with an epileptogenic lesion in the temporal lobe other than HS were classified as lesional TLE. Probands with non-lesional TLE were further classified into MTLE, lateral TLE and non-localized TLE. Twins with auditory auras or auditory sensory symptoms during seizures were classified as having lateral TLE. The twins were classified as having non-localized TLE when no further electroclinical localizing features beyond the temporal lobe were present. The diagnosis of MTLE was made according to previously defined electroclinical criteria indicating mesial temporal seizure onset (Box 1).^2^

Box 1 Clinical criteria used for the diagnosis of mesial temporal lobe epilepsy^2^ Presence of one or more of the following semiological features at seizure onset referable to the mesial temporal lobe:

- déjà vu;
- stereotyped flashbacks of a past event;
- rising epigastric/visceral sensation;
- stereotyped olfactory or gustatory hallucinations

Or

• mesial temporal lobe onset of seizures as recorded via intracranial EEG recording

And

• absence of an epileptogenic lesion other than hippocampal sclerosis on neuroimaging

In the MTLE cohort, twins were further subclassified into MTLE with or without HS, based on the presence or absence of HS as confirmed by neuroimaging and/or histopathology. Diagnosis of HS on neuroimaging was made based on visual inspection. MRI for all MZ twin pairs with HS was independently reviewed by an epileptologist with neuroimaging expertise (G.D.J.) and a neuroradiologist (G.F.). Where there were discrepancies, discordant interpretations were conferred for consensus.

### Zygosity assessment

In same-sex twin pairs, zygosity was determined using a validated zygosity questionnaire with more than 95% accuracy,^16^ and confirmed by genotyping. DNA from either blood, saliva or buccal samples were sent to the Australian Genome Research Facility for zygosity testing using 10 highly polymorphic short tandem repeat markers (AMEL for sex identification, CSF1PO, D13S317, D16S539, D21S11, D5S818, D7S820, TH01, TPOX, vWA), with a 0.00016 average probability of a dizygotic pair being identical at all the loci tested.^17^

### Concordance

Twin pairs where both individuals were diagnosed with TLE were designated as concordant for TLE. Similarly, twin pairs were concordant for HS when both individuals in the pair had a diagnosis of HS.

Casewise concordance (*P_c_*), defined as the probability that a twin individual is affected given that the co-twin is affected, was estimated as P_c_ = 2n_c_/(2n_c_ + n_d_), where n_c_ is the number of concordant pairs and n_d_ the number of discordant pairs.^18^ Casewise concordances for TLE and HS were calculated in MZ twin pairs and compared with those in the DZ twin pairs to determine the genetic contribution to TLE and HS, respectively.

The temporal dimension of concordance for TLE was studied using Kaplan–Meier survival analysis. Using survival analysis, we plotted the probability of TLE diagnosis in the co-twin at various time points against the time of discordance, defined as the time lag between TLE diagnoses in the index twin and the co-twin.

### Statistical analyses

Statistical analyses were performed using R version 4.3.0 (R Core Team 2025, https://www.R-project.org/). The differences in the number of concordant pairs between the MZ and DZ twins for TLE and each TLE subtype, as well as the differences in clinical characteristics between the MTLE-HS and MTLE without HS twin pairs, were analysed using a two-sided Fisher's exact test. Bonferroni adjustments were applied to account for multiple comparisons, as appropriate. An adjusted *P* < 0.05 was considered statistically significant.

The probability of TLE diagnosis in the co-twin was estimated using the Kaplan–Meier method with the survminer R package.^19^ Log-rank sum test was used to determine the distribution differences between MZ and DZ twin pairs.

### Molecular testing

Molecular testing was not performed for the whole cohort. Twin individuals were tested when they had epilepsy syndromes suspected to be associated with specific genes or if they were included in larger collaborative studies where genomic sequencing was performed. Molecular testing was performed on individuals where DNA samples (blood, saliva) were available. The molecular testing methods included single-gene sequencing, single nucleotide polymorphism (SNP) genotyping, epilepsy gene panel analysis, whole-exome sequencing (WES) and whole-genome sequencing (WGS). Bioinformatic analysis and variant calling were performed using established germline pipelines. Variants were classified according to the American College of Medical Genetics and Genomics (ACMG) guidelines.^20^ Pathogenic and likely pathogenic variants were validated with Sanger sequencing or digital droplet polymerase chain reaction.

## Results

### Twin cohort characteristics

Our cohort comprised 160 individuals (104 female, 56 male) from 80 twin pairs (47 MZ, 33 DZ pairs). Of these, 30 twin individuals had a history of febrile seizures, with 73% (22/30) of these twins later developing TLE. A total of 97 twin individuals in our cohort were diagnosed with TLE. Thirteen twin individuals had a history of prolonged febrile or afebrile seizures.

The mean age of epilepsy onset was 16.9 years [median age of onset 15 years, interquartile range (IQR) 7–23 years]. Ninety-two twin individuals were recruited by referral, while 68 individuals were recruited from a community-based register.

### Casewise concordances for TLE

**Figure 1:**
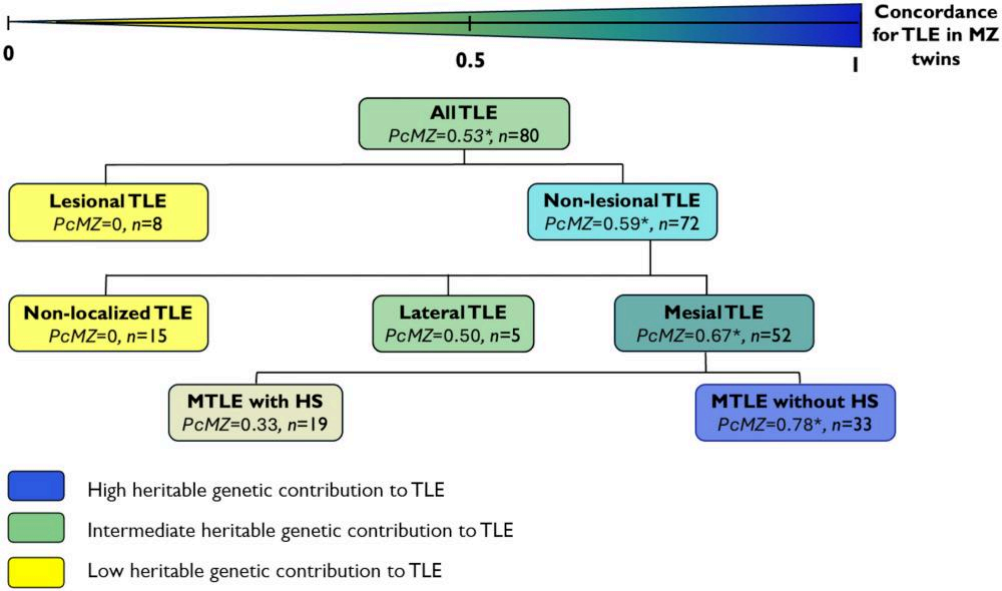
**Genetic contributions to TLE subtypes based on casewise concordances for TLE**. *PcMZ*– casewise concordance for TLE in MZ twins. This was calculated as P_c_ = 2n_c_/(2n_c_ + n_d_), where n_c_ is the number of twin pairs concordant for TLE and n_d_ is the number of twin pairs discordant for TLE. None of the DZ twin pairs were concordant for TLE. The difference in the number of concordant pairs between MZ and DZ twin pairs for TLE and each TLE subtype was analysed using a two-sided Fisher's exact test. *Adjusted *P* < 0.05 (adjustment for multiple comparisons) was considered statistically significant. DZ = dizygotic; HS = hippocampal sclerosis; MZ = monozygotic; MTLE = mesial temporal lobe epilepsy; TLE = temporal lobe epilepsy.

Figure 1 shows the casewise concordances (*PcMZ*) for TLE and its subtypes in MZ twins. Details on the proportion of MZ twins concordant for TLE within each subtype, along with comparisons to DZ twin concordances (*PcDZ*), are available in Supplementary Table 1. The casewise concordance for TLE was significantly higher in MZ twins than in DZ twins, with no concordant pair observed in the latter (*PcMZ* = 0.53, *PcDZ* = 0; *P* < 0.05). Notably, the increased concordance in MZ twins was confined to the non-lesional TLE cases (*PcMZ* = 0.59, *PcDZ* = 0; *P* < 0.05), as there were no concordant cases among MZ or DZ twins with lesional TLE (Fig. 1; Supplementary Table 2).

Within the non-lesional TLE group, the concordance seen in MZ twins was driven by the MTLE cases (*PcMZ* = 0.67, *PcDZ* = 0; *P* < 0.05). When the MTLE cases were further subclassified based on the presence or absence of HS, an even higher MZ twin concordance for TLE was observed in the MTLE without HS twins (*PcMZ* = 0.78, *PcDZ* = 0; *P* < 0.05).

In contrast to the high MZ concordance for TLE observed in the MTLE without HS twins, the concordance for TLE was much lower in MZ twins with MTLE-HS. There were 19 twin pairs (10 MZ, 9 DZ) where one or both twins had a diagnosis of MTLE-HS. Only two MZ pairs were concordant for TLE; in one pair both twins had HS, while in the other pair, only one twin had HS. No DZ pair was concordant for TLE.

Five twin pairs were classified as having lateral TLE (3 MZ, 2 DZ); four of the twin pairs had sporadic lateral TLE and one discordant DZ pair had familial lateral TLE. Of the three MZ twin pairs with lateral TLE, one pair was concordant for TLE. None of the DZ twin pairs with lateral TLE were concordant.

**Figure 2:**
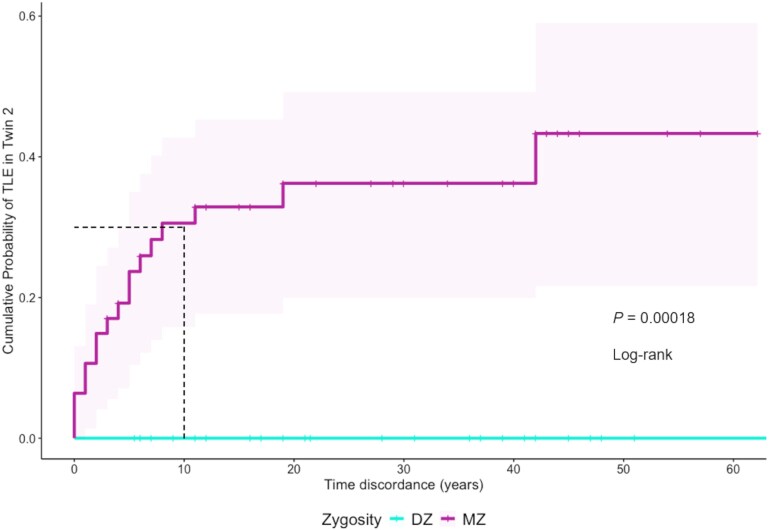
**Probability of co-twin being diagnosed with TLE at various time points after the TLE diagnosis made in the index twin, stratified according to zygosity *n* = 80**. The shaded colour regions define the 95% confidence intervals. Coloured vertical dashes along each survival curve denote the most recent age of co-twin known without a diagnosis of TLE (censored observations). The probability of TLE diagnosis in co-twin 10 years after the index twin's diagnosis for each group is indicated by black dashed lines. DZ = dizygotic; MZ = monozygotic; TLE = temporal lobe epilepsy.

In the survival analysis, the probability of an MZ co-twin being diagnosed with TLE within 10 years of the index twin's diagnosis (i.e. 10-year time discordance) was 30.6% (95% confidence interval): 15.8–42.7%; *P* < 0.05). Additionally, among the concordant MZ twins, most MZ twins (15 out of 17) were concordant for TLE within 10 years (Fig. 2).

### Casewise concordance for HS

**Figure 3:**
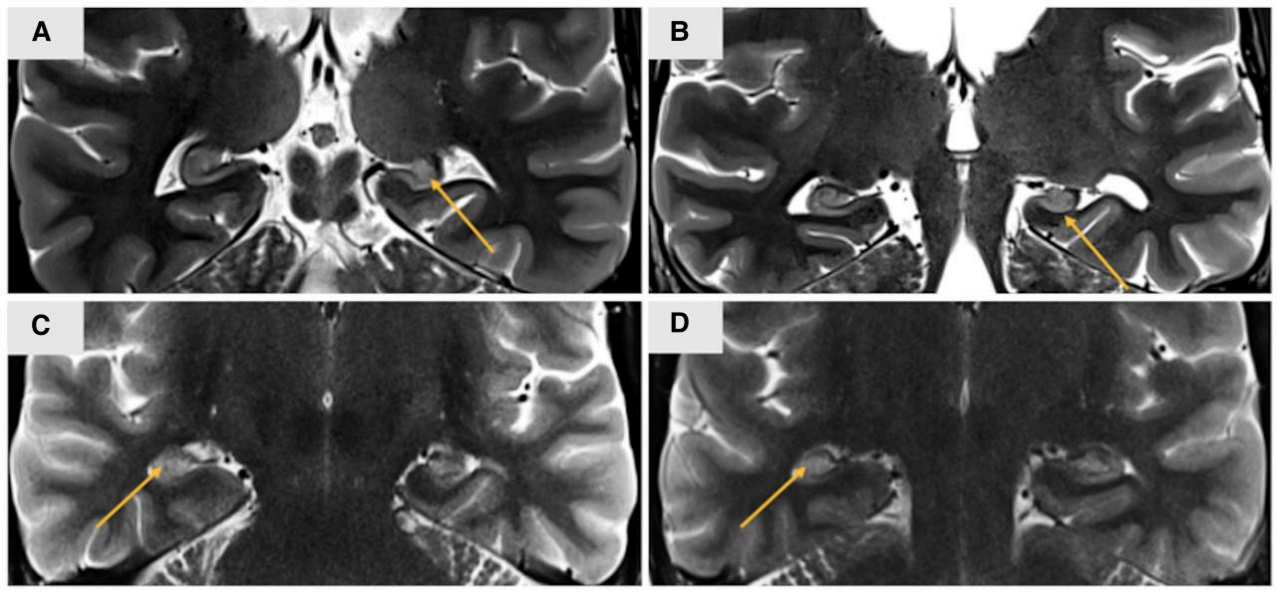
**MRI coronal T2-weighted images for two monozygotic twin pairs with neurofibromatosis type 1 who were concordant for hippocampal sclerosis**. (**A** and **B**) Pair 1. (**C** and **D**) Pair 2. In each case, both hippocampi were abnormal; however, there was a lateralized emphasis. The hippocampus ipsilateral to the TLE in each twin met the diagnostic criteria for hippocampal sclerosis (HS), while the contralateral hippocampus displayed a high T2 signal. Pair 1: (**A**) left HS (arrow). This twin also had an increased T2 signal in the posterior body of the right hippocampus (not shown). (**B**) Left HS (arrow) and high T2 signal in the right hippocampus. Pair 2: (**C**) right HS (arrow) and high T2 signal in the left hippocampus. (**D**) Right HS (arrow) and increased T2 signal in the left hippocampus. TLE = temporal lobe epilepsy.

Two MZ twin pairs had epilepsy and were concordant for HS (Fig. 3). In one pair (Pair 1, Fig. 3A and B), both twins had a diagnosis of TLE, whereas, in the second MZ pair (Pair 2, Fig. 3C and D), the proband had TLE, while the co-twin did not fulfil criteria for TLE and was regarded as having ‘unclassified’ epilepsy. Both twin pairs have a clinical and genetic diagnosis of neurofibromatosis type 1 (NF1) with pathogenic germline variants in *NF1* (*NF1* c.3826 C > T p.Arg1276Ter and *NF1* c.2125T > C p.Cys709Arg).

### MTLE without HS twin pairs versus MTLE-HS twin pairs

We compared the MTLE-HS twin pairs with the MTLE without HS pairs, to identify differences in the clinical characteristics of the affected twins between the two groups (Table 1).

**Table 1:** Clinical characteristics of MTLE-HS twin pairs compared with MTLE without HS pairs

Clinical characteristics	MTLE-HS (*N* = 19), *n* (%)	MTLE without HS (*N* =33), *n* (%)	Adjusted *P*-value*
Family history of epilepsy	3 (16%)	17 (52%)	0.10
Family history of TLE	1 (5%)	12 (36%)	0.10
Family history of febrile seizures	2 (11%)	2 (6%)	1
Antecedent febrile seizures	10 (53%)	6 (18%)	0.08
Antecedent prolonged febrile seizures	6 (32%)	0 (0%)	0.01

MTLE = mesial temporal lobe epilepsy; HS = hippocampal sclerosis; TLE = temporal lobe epilepsy.

^*^Adjusted *P* < 0.05 was considered statistically significant.

Antecedent events were identified in 63% of those with MTLE-HS (12/19; 6 MZ, 6 DZ), with 53% (10/19; 6 MZ and 4 DZ) having a history of antecedent febrile seizures. Among these, six twins (4 MZ, 2 DZ) had prolonged febrile seizures. The other two DZ twins with MTLE-HS had different antecedent events: one had anoxic brain injury and the other had prolonged afebrile seizures. In contrast, only 18% (6/33) of the MTLE without HS twins had an identifiable antecedent event, which in all cases was a history of febrile seizures. Notably, none of the MTLE without HS twins had antecedent prolonged febrile or afebrile seizures.

MTLE-HS twins demonstrated an earlier onset of TLE with a median age of 7 years (IQR 2–19.5), compared with MTLE without HS twins, who had a median onset age of 20 years (IQR 12–26). Additionally, a family history of epilepsy and TLE was more common in the MTLE without HS twins compared with those with MTLE-HS; nonetheless, no significant differences were noted between the two groups with respect to these two variables.

### Twin-twin comparison for antecedent events in MTLE-HS twins

**Figure 4:**
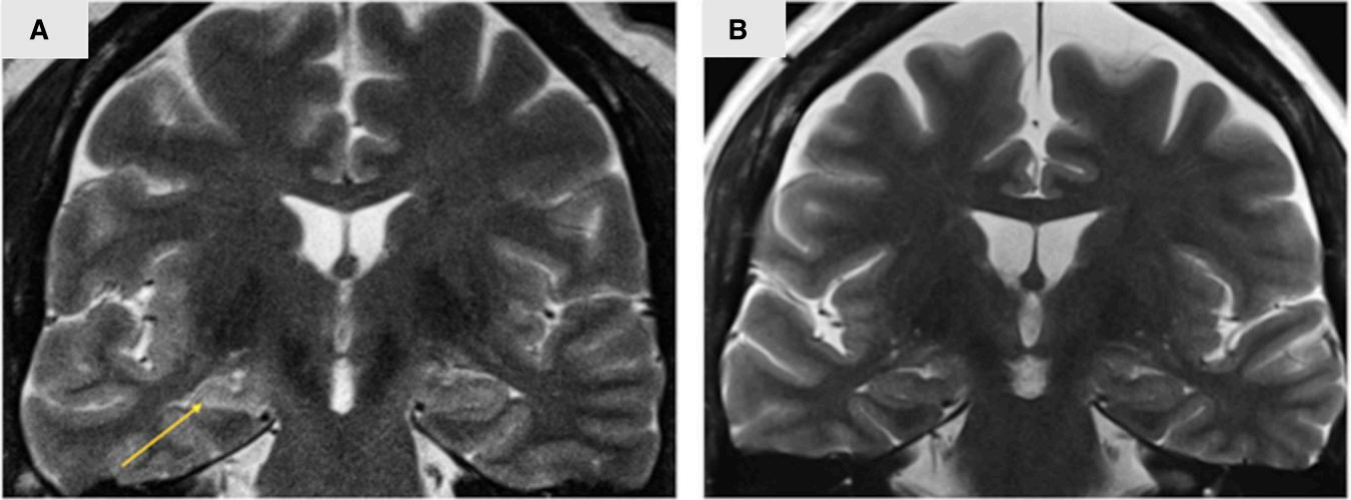
**MRIs of a monozygotic twin pair concordant for MTLE and antecedent febrile seizure but discordant for hippocampal sclerosis**. Right hippocampal sclerosis (HS) was demonstrated only in twin (**A**) with a history of prolonged febrile seizures. (**B**) Similarly positioned image from co-twin with no evidence of HS but with generalized parenchymal loss of undetermined cause. The MRI in Fig. 4A was taken at age 41 years before her epilepsy surgery, while her co-twin's MRI in Fig. 4B was taken at age 54 years. This twin pair was not included in our previous study of monozygotic twins with HS.^11^ MTLE = mesial temporal lobe epilepsy.

We compared the 10 MZ pairs where at least one twin had HS to assess acquired differences between affected and unaffected individuals. Of these, eight pairs were discordant for HS and two were concordant. The two concordant pairs had both HS and NF1, with no antecedent insults identified. Among the discordant pairs, two pairs had no identifiable antecedent events, while in two other discordant pairs, only the affected proband with HS had febrile seizures. In the remaining four discordant pairs, both twins had febrile seizures, but HS was present only in the twin with prolonged febrile seizures. This included one twin pair where both twins had febrile seizures and MTLE, but HS was observed only in the twin with prolonged febrile seizures (Fig. 4). Additionally, another pair shared an *SCN1B* c.363C > G, p.Cys121Trp variant as part of a family with Genetic Epilepsy with Febrile Seizures Plus (GEFS+), which accounted for febrile seizures in both twins.^12^

### Molecular analysis

Molecular testing was performed on 55 (69%) twin pairs: 28 had one twin tested, while 27 had both twins tested, resulting in a total of 82 individuals tested. Among these individuals, 21 underwent single-gene sequencing, 28 had SNP genotyping, 4 had focal epilepsy gene panels, 35 underwent WES and 4 had WGS. Detailed information regarding the types of molecular testing conducted for all twins in this cohort is provided in Table 2.

**Table 2:** Molecular testing for twins in the cohort according to TLE subtypes

TLE subtype (number of twin pairs)	Molecular testing			Type of molecular testing (*n*)^a^		Positive findings (ACMG classification)
	None	One twin	Both twins	Twin 1	Twin 2	
Lesional (8)	1	6	1	SNP (3)	Panel (1)	None
				Panel (1)		
				WES (2)		
				WGS (1)		
Non-localized (15)	10	1	4	WES (4)	SNP (2)	None
				WGS (1)	WES (1)	
				–	WGS (1)	
Lateral (5)	2	0	3	Single gene (3)	Single gene (3)	None
MTLE without HS (33)	10	12	11	SNP (4)	SNP (3)	^b^ *NPRL3* c.1376_1377insAC, p.Ser460Profs*20 Paternally inherited (pathogenic, PVS1 PM2, PP1, PP4) Present in affected twin and affected father
				Single gene (7)	Single gene (4)	
				WES (12)	Panel (1)	
				–	WES (3)	
MTLE with HS (19)	2	9	8	SNP (2)	SNP (4)	^c^ *SCN1B* c.363C>G, p.Cys121Trp Paternally inherited (pathogenic, PS4, PS3, PM1, PP1) Present in both twins and affected father
				Single gene (2)	Single gene (2)	
				WES (12)	Panel (1)	
				WGS (1)	WES (1)	
						^b^ *NF1* c.3826 C>T, p.Arg1276Ter *De novo* (pathogenic, PVS1, PS4, PM2)
						^d^ *NF1* c.2125T>C, p.Cys709Arg Maternally inherited (pathogenic, PS1, PM2, PP3, PP1, PP2, PP4) Present in both twins and affected mother
Total (%)	25 (31%)	28 (35%)	27 (34%)	–		

A total of 28 twins have had SNP genotyping done. Of these, 10 were not included in the table as they were included in the other molecular testing counts (e.g. WES, WGS). ACMG = American College of Medical Genetics and Genomics; HS = hippocampal sclerosis; MTLE = mesial temporal lobe epilepsy; TLE = temporal lobe epilepsy; SNP = single nucleotide polymorphism; WES = whole-exome sequencing; WGS = whole-genome sequencing.

^a^Molecular testing methods in this cohort include single nucleotide polymorphism genotyping (denoted as SNP in the table), single-gene sequencing (denoted as single gene in the table), epilepsy gene panel analysis (denoted as panel in the table), WES and WGS.

^b^Both *NPRL3* c.1376_1377insAC, p.Ser460Profs*20 and *NF1* c.3826 C>T, p.Arg1276Ter pathogenic variants were detected using single gene sequencing.

^c^The *SCN1B* c.363C>G, p.Cys121Trp pathogenic variant is shared by the monozygotic twins and father, which accounted for febrile seizures in both twins and their father. This variant was detected through WES.

^d^The *NF1* c.2125T>C, p.Cys709Arg pathogenic variant was identified through WES.

Four positive findings were identified: (i) a previously reported paternally inherited *NPRL3* frameshift variant (*NPRL3* c.1376_1377insAC, p.Ser460Profs*20) was identified in a proband with MTLE without HS, which segregated with epilepsy within the family^21^; (ii) in the above-mentioned MZ twin pair that was concordant for febrile seizures but discordant for MTLE-HS, a previously reported paternally inherited *SCN1B* c.363C > G, p.Cys121Trp variant was found in both twins as part of a GEFS+ family^12^; and (iii) two twin pairs that met the clinical diagnostic criteria for NF1 and were concordant for HS had pathogenic *NF1* variants (*NF1* c.3826 C > T p.Arg1276Ter and *NF1* c.2125T > C p.Cys709Arg) identified.

## Discussion

Our findings indicate differences in the magnitude of concordance for TLE among MZ twins across the various TLE subtypes, suggesting that there are varying degrees of germline genetic contribution associated with each subtype (Fig. 1). In the 10 MZ pairs where at least one twin had HS, our data reinforce the well-recognized association of prolonged febrile seizures with MTLE-HS. We uniquely found two MZ twin pairs concordant for HS. Remarkably, both pairs carry a pathogenic germline *NF1* variant and the clinical diagnosis of NF1.

A heritable genetic contribution to TLE was only observed in our non-lesional twins, as there were no concordant twins with lesional TLE. However, this does not exclude a genetic contribution to lesional TLE, given that twin studies are not sensitive to detecting somatic (post-zygotic) mutations. Therefore, while our data suggest germline variants are unlikely in lesional TLE, the possibility of somatic mutations remains, supported by increasing evidence from post-surgical tissue samples, which was not interrogated in this study.^22-24^

Our twin method allows us to explore the relative germline genetic contribution to non-lesional TLE subtypes, which is difficult to do in a non-twin population. In lateral TLE, our findings indicate a moderate heritable genetic contribution. Autosomal dominant families with lateral TLE or epilepsy with auditory features have been described, with pathogenic variants in *LGI1* and *RELN* genes found in 50% of these families.^8,25,26^ Previous studies have indicated that most monogenic causes of lateral TLE are associated with familial cases, and pathogenic variants in *LGI1* are identified in less than 5% of sporadic lateral TLE cases.^25-27^ Within our cohort, only one twin pair had familial lateral TLE, while the other four pairs had sporadic lateral TLE. Molecular testing for *LGI1* variants was conducted for three twin pairs, including the familial pair; however, no pathogenic variants were identified.

The heritable genetic contributions to TLE were primarily driven by MTLE without HS. Among the cases studied, 52% of MTLE without HS individuals had a family history of epilepsy, with 36% having a family history of TLE, reinforcing the notion of germline genetic factors influencing this subtype. This finding aligns with the recognized familial epilepsy syndrome of FMTLE, which was first described using twins.^7^ Pedigree analysis of FMTLE families suggests that dominant inheritance is rare in these cases.^2^ Pathogenic variants in GATOR1 complex genes have been identified, but only in a minority of FMTLE cases.^28^ Twenty-two of our MTLE without HS twins underwent molecular testing; a previously reported pathogenic *NPRL3* variant was identified in one discordant dizygotic pair.^21^ Recent research has highlighted the role of common genetic variation in FMTLE, as evidenced by greater focal epilepsy polygenic risk observed in 227 FMTLE cases compared with population controls.^3^ This finding supports a polygenic basis for FMTLE, which likely accounts for a significant proportion of the germline genetic contribution observed in MTLE without HS.^3^

In contrast, in the MTLE-HS twins, we found prolonged febrile seizures to be prevalent with a much lesser germline genetic contribution. This suggests that non-genetic factors play a major role in this subtype, which is the most common histopathological finding among adults with drug-resistant epilepsy undergoing surgery.^29^ Our twin data indicate that while MTLE with and without HS may present similar clinical features aside from subtle differences in seizure duration,^30^ their aetiology and genetic architectures differ.

Evidence for the role of prolonged febrile seizures in HS was first proposed by Falconer in the 1960s.^31^ Since then, both prolonged febrile and afebrile seizures have been shown to contribute to the development of HS.^11,32,33^ In the prospective FEBSTAT (Consequences of Prolonged Febrile Seizures in Childhood) study, hippocampal signal abnormalities were observed in 10% (22/226) of children following febrile status epilepticus, and 70% (10/14) of those with abnormal signal who returned for a follow-up MRI developed HS.^32,34^ Notably, some of these children had pre-existing subtle hippocampal abnormalities, possibly predisposing them to febrile status epilepticus; indicating that both mechanisms are likely operative in the development of HS in this cohort.^32,34^

Our twin data further reinforces the association between prolonged febrile seizures and HS. In our cohort of MZ twins, prolonged febrile seizures were observed only in the probands with HS. Furthermore, even in MZ pairs where both twins had febrile seizures, only the twin with a history of prolonged febrile seizures developed HS. This suggests that while there may be a genetic predisposition to febrile seizures, such as the shared germline *SCN1B* variant identified in one of the MZ pairs accounting for the concordance for febrile seizures, the development of prolonged febrile seizures—and subsequent HS—may be influenced by additional factors, resulting in only one twin with HS. The prolonged nature of these seizures is likely to have conferred an additional ‘hit’ resulting in HS.

Other acquired factors associated with HS include head trauma, encephalitis and amphetamine use, which were not observed in our twins.^35-37^ In our cohort, one of the affected twins had a history of anoxic brain injury.

**Figure 5:**
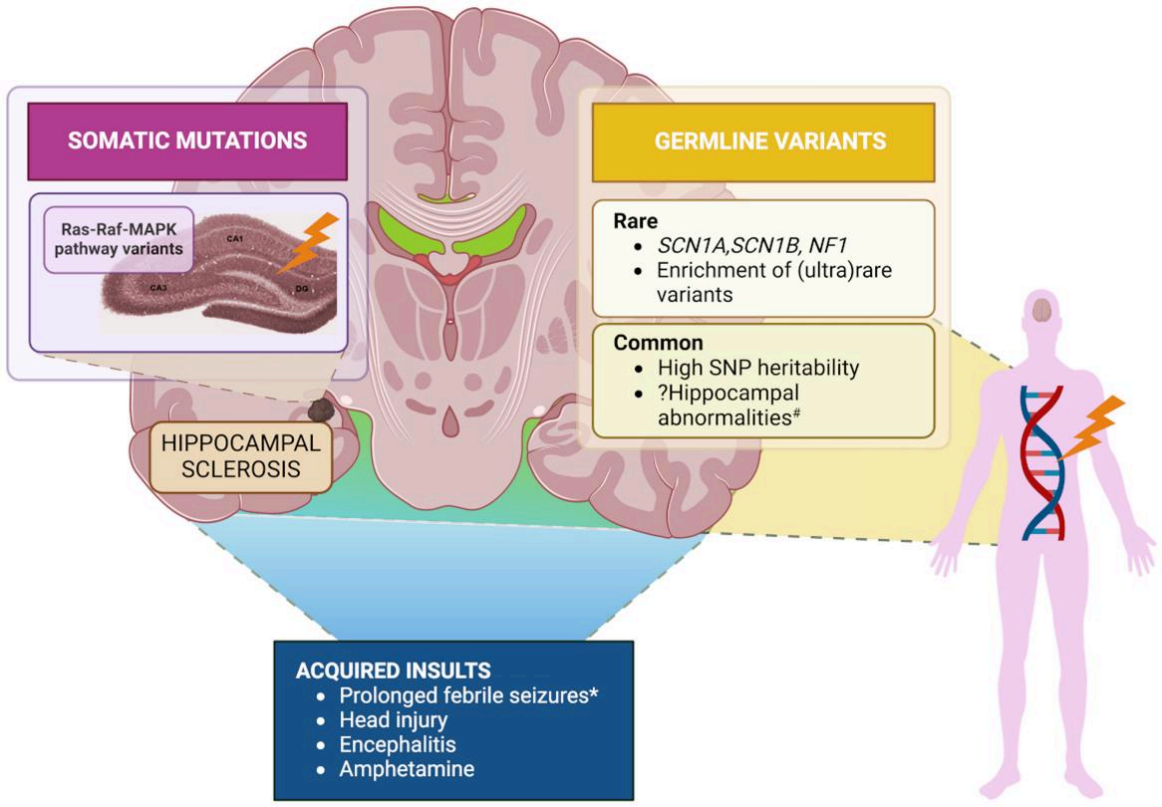
**Genetic contributions to hippocampal sclerosis (HS) and the interaction between acquired and genetic factors in HS**. The genetic contributions to HS can be categorized into two main types: germline variants, which can be either rare or common, and somatic mutations affecting the Ras/Raf/Mitogen-activated protein kinase (MAPK) pathway within the hippocampus.^22^ The mechanisms underlying HS are likely influenced by one or more of these genetic factors, as well as interactions with acquired insults (indicated by the shaded green area) that also contribute to the development of HS. *Genetic factors, e.g. variants in *SCN1A* and *SCN1B* predispose to febrile seizures and very rarely prolonged febrile seizures.^12,38^  ^#^Hippocampal abnormalities have been observed in families with HS, suggesting a potential association between these abnormalities and HS.^39-41^ SNP = single nucleotide polymorphisms. Created in BioRender. Dang, Y. (2025) https://BioRender.com/k71a861.

Regarding genetic contributions to HS, multiple emerging lines of evidence suggest this (Fig. 5). Rare familial cases of HS have been documented, often with antecedent febrile seizures.^39,42^ Additionally, family studies have identified hippocampal abnormalities and an increased prevalence of febrile seizures and epilepsy in relatives of individuals with MTLE-HS.^39-41^

More recent studies have explored the role of rare and common germline variants in MTLE-HS. Enrichment of rare germline variants in fragile X mental retardation protein-related genes,^43^ constrained genes intolerant to loss-of-function variants and genes encoding voltage-gated cation channels were found in cohorts with MTLE-HS.^43,44^ Additionally, a meta-analysis of a genome-wide association study (GWAS) suggested that common variation in *SCN1A* increases the risk for MTLE-HS and febrile seizures.^38^ Although no genome-wide significant loci were identified for focal epilepsy with HS (*n* = 1375) in the 2023 epilepsy GWAS, a single nucleotide polymorphism-based heritability of 47% (*n* = 1260) was observed in focal epilepsy with HS supporting a contribution of common variation to HS.^45^

Previous small studies of MZ twins with HS have shown discordance for HS,^11,46,47^ which does not support a genetic contribution. However, our larger data set refines this view with the observation of two MZ twin pairs who were concordant for HS. Both pairs had germline variants in *NF1*, indicating that this gene is relevant to the development of HS. Of note, a number of considerations suggest distinct characteristics in NF1-associated HS compared with other HS cases. Firstly, while HS is relatively common in epilepsy surgery series, its association with epilepsy in NF1 is rare, with reports indicating its presence in only 0.2%–3% of surgical cohorts of NF1 patients.^48^ Secondly, in our study, both MZ twin pairs with NF1 and HS exhibited contralateral hippocampal hyperintensities, a feature not seen in other twins with HS in our cohort. Hippocampal hyperintensities are identified in 60%–80% of NF1 patients and have been linked to HS in NF1; however, they have not been observed in other HS cases.^49-52^ Lastly, we observed that NF1-associated HS is not linked with antecedent events, such as prolonged febrile seizures, which are commonly associated with HS. Our MZ twins with NF1 and HS did not report any antecedent events, consistent with the limited literature regarding their role in NF1-associated HS.^48,53,54^

Although the mechanisms for HS in NF1 remain poorly understood, our observation aligns with recent evidence of pathogenic somatic variants in the Ras/Raf/Mitogen-activated protein kinase (MAPK) pathway genes, including *NF1,* in resected hippocampi of refractory TLE cases with HS.^22^ Further, *NF1* somatic variants were identified in the resected hippocampal tissue of two unrelated individuals from that study with drug-resistant MTLE-HS, both of whom had a clinical diagnosis of NF1 and germline variants in the *NF1* gene.^22^ This finding underscores the ‘double-hit’ mechanism, previously described in NF1-associated conditions,^55^ extending its relevance to MTLE-HS in the context of NF1.

Six twins in this cohort did not meet the specified clinical criteria for MTLE. However, they were classified as MTLE-HS based on the finding of HS on imaging and/or histopathology. Four of these twins experienced autonomic features during seizures. Although this feature was not in our pre-specified diagnostic criteria for MTLE, it has been described as a feature of MTLE-HS in the 2022 ILAE classification of epilepsy syndromes.^13^ The remaining two twins did not experience any aura, making it challenging to localize seizures to the mesial region, due to the low likelihood of localization using scalp EEG. In these cases, a mesial temporal lobe seizure onset was inferred based on the presence of HS, and the absence of clinical features indicating a different localization. Additionally, 15 twin pairs (6 MZ, 9 DZ) were classified as non-localized TLE, as further electroclinical localizing features were absent. However, we acknowledge that some of these twins may have a mesial onset of seizures that could not be confirmed with the available data.

Ascertainment bias is a limiting factor in most twin studies, as concordant twins, particularly concordant MZ twins, are more likely to be recruited by referral than from a registry-based sample.^9^ In our study, we included twins from both community registries and referrals to minimize selection bias. Our cohort comprised equal proportions of MZ twins from both recruitment sources, with 20 out of 34 pairs (59%) from the registry-based sample and 27 out of 46 pairs (59%) from the referral-based sample. Additionally, the casewise concordance for TLE in MZ twins was consistent across both samples, with a *PcMZ* of 0.54. This consistency underscores the validity of our findings, as it applies to both twins recruited from the community-based registry and the broader cohort, thereby reducing the impact of ascertainment bias.

As the twins were studied over three decades, molecular analysis was not systematically performed for all twins with available DNA. Of the 55 twin pairs that underwent molecular testing, only 39 individuals had either WES or WGS. Consequently, additional germline variants relevant to the TLE subtype may have been missed.

## Conclusion

Our study highlights the differential genetic contributions to TLE subtypes. A strong heritable genetic component is demonstrated in non-lesional TLE, particularly in MTLE without HS. A germline genetic contribution is also observed in lateral TLE but is absent in lesional and non-localized TLE. In MTLE-HS, our data support the role of both genetic and non-genetic factors. Notably, *NF-1* has been identified as a gene relevant to HS. Considering the rarity of HS descriptions in NF1 cases, with only 20 cases reported in the literature,^48,56^ the identification of multiple affected individuals with both NF1 and HS in this small group suggests a non-random association, warranting further detailed investigation. Future clinical and molecular studies focused on understanding the contribution of hippocampal somatic *NF1* mutations, alongside germline *NF1* variants, to the pathophysiology of HS would be invaluable.

## Supplementary Material

awaf209_Supplementary_Data

## Data Availability

The data that support the findings of this study are available from the corresponding author, upon reasonable request.
