# Supplementary material for: Twins with temporal lobe epilepsy: genetic contributions to hippocampal sclerosis and other subtypes
Source: Brain. 2025 Jun 18;148(11):3989–99. doi: 10.1093/brain/awaf209 (PMC12588710; doi:10.1093/brain/awaf209)
Supplement: awaf209_Supplementary_Data [file awaf209_supplementary_data.pdf]

Supplementary material.

Supplementary Table 1 Casewise concordances for TLE and TLE subtypes (as shown in Figure 1)

	Proportion of MZ or DZ twins concordant for TLE		Casewise Concordance for TLE		Adjusted *p* value*
**Clinical Characteristics**	**MZ**	**DZ**	***PcMZ***	***PcDZ***	
All TLE	17/47	0/33	0.53	0	<0.05
**TLE Subtypes**					
Lesional TLE	0/6	0/2	0	0	1
Non-lesional TLE	17/41	0/31	0.59	0	<0.05
Non-localized TLE	0/6	0/9	0	0	1
Lateral TLE	1/3	0/2	0.50	0	1
Mesial TLE	16/32	0/20	0.67	0	<0.05
MTLE with HS	2/10	0/9	0.33	0	1
MTLE without HS	14/22	0/11	0.78	0	<0.05

PcMZ or *PcDZ* – casewise concordance for TLE in MZ and DZ twins respectively. This was calculated as $P_c = 2n_c/(2n_c + n_d)$, where n_c is the number of twin pairs concordant for TLE and n_d is the number of twin pairs discordant for TLE.
*Adjusted $p<0.05$ (adjustment for multiple comparisons) was considered statistically significant. The difference in the number of concordant pairs between MZ and DZ twin pairs for TLE and each TLE subtype was analysed using a two-sided Fisher's exact test.
Abbreviations: DZ, dizygotic; HS, hippocampal sclerosis; MZ, monozygotic; MTLE, mesial temporal lobe epilepsy; TLE, temporal lobe epilepsy.

Supplementary Table 2 Types of lesions in twins with lesional TLE (6 MZ, 2 DZ)

Type of lesion	Number of twins and zygosity
**Focal cortical dysplasia**	2 DZ
**Schizencephaly and periventricular nodular heterotopia**	1 MZ
**Periventricular nodular heterotopia**	1 MZ
**Ganglioglioma**	2 MZ
**Arteriovenous malformation**	1 MZ
**Poorly characterized lateral temporal lobe lesion (CT Brain)**	1 MZ

In the lesional TLE twins, none of the twins were concordant for TLE. Six of the twins had lateral TLE whereas two had mesial TLE.
Abbreviations: DZ, dizygotic; MZ, monozygotic; TLE, temporal lobe epilepsy.
